# A cross-sectional study on self-reported physical and mental health-related quality of life in rheumatoid arthritis and the role of illness perception

**DOI:** 10.1186/s12955-018-1064-y

**Published:** 2018-12-19

**Authors:** Carolin Berner, Ludwig Erlacher, Karl Heinrich Fenzl, Thomas E. Dorner

**Affiliations:** 1 Sozialmedizinisches Zentrum-Süd, Department of Rheumatology and Osteology, Kaiser Franz Josef Hospital, Vienna Kundratstrasse 3, 1100 Vienna, Austria; 2 Karl Landsteiner Institute for Autoimmune Diseases and Rheumatology, Vienna, Austria; 3 Department for Social and Preventive Medicine, Centre for Public Health, Medical University of Vienna, Vienna, Austria

## Abstract

**Background:**

Psychosocial models including illness perception might explain individual differences in health-related quality of life (HRQoL) and daily functioning in chronically ill patients. The aim of this study was to assess the association of illness perception among rheumatoid arthritis (RA) patients with physical and mental HRQoL, adjusted for demographic variables, clinical variables and social support.

**Methods:**

A cross-sectional study conducted at a Viennese rheumatology outpatient clinic on 120 RA patients. Participants completed questionnaires on demographic and clinical characteristics, HRQoL (SF-36 Questionnaire), illness beliefs (Brief Illness Perception Questionnaire) and social support (Social Support Scale-8). Analyses were performed with multivariate linear regression.

**Results:**

The mean physical was lower (38.38) than the mean mental SF-36 summary score (46.94). In univariate analysis, all domains of illness perception except belief in a chronic disease course were associated with physical and mental HRQoL. In multivariate analyses, illness perception accounted for 51% of variance in physical HRQoL. A stronger belief in the consequences of RA (*consequences,* β = − 0.33) and a stronger belief in repeated disease recurrence (*timeline cyclical,* β *= − 0 .31*) were significantly associated with physical HRQoL in the fully adjusted model. Illness perception accounted for 45% of variance in mental HRQoL. *Emotional representation* (β = − 0 .27) and fatigue (β = − 0 .36) were significantly associated with mental HRQoL in the fully adjusted model.

**Conclusion:**

This study highlights the importance of RA patients’ beliefs about their illness and symptoms in relation to HRQoL. Identification of patients’ perception of RA may be a way to positively influence disease outcomes such as quality of life as illness perception is amenable to intervention.

## Introduction

Rheumatoid arthritis (RA) is a chronic systemic disabling condition with a global prevalence of an estimated 0.2–1.0% [1] that can lead to impairment in activities of daily living and work productivity and compromise the overall well-being of RA patients [2]. In RA, the presentation and course of the disease are individual for every patient. The severity of the disease can be confined to mild articular affection or manifested as severe, multisystem disease. Also, individuals’ perceptions of single symptoms differ and have been attributed to individual differences in adaptation to rheumatoid arthritis [3, 4]. Despite significant improvements in the medical management, treatment and prognosis of RA, it is common for patients to experience deficits in physical and mental health functioning [5, 6]. The burden of disease in RA depends on both emotional and physical components and the respective impact on overall well-being may vary in individual patients. As specific RA therapy usually targets specific outcomes (e.g. disease activity), the evaluation of its success is mostly seen from the clinical view and rarely includes a patient’s overall health-related quality of life (HRQoL) [7, 8]. Therapeutic interests thus increasingly shift from merely symptomatic treatment to restoring, or at least improving, HRQoL [8].

Despite similar levels of disease activity and severity, rheumatologists have observed individual variations in HRQoL. Therefore, more and more emphasis is put on other factors in addition to a strict medical/symptom-related model. Social support, for example, has a substantial effect on QoL [9] and previous research has shown that social support has beneficial effects on RA patients [10, 11].

Also, psychosocial models were discussed as a framework to explain these individual differences in HRQoL and daily functioning in chronically ill patients [5, 12]. An individual’s reaction and adaptation to a health threat such as a chronic disease depends on the individual’s cognitive and emotional representation of this threat in their mind [13]. This psychological factor, which is considered to be an important determinant of perception [14].

In the Common-Sense Model (CSM) of Howard Leventhal and colleagues, illness perception is the key construct that suggests that people have personal beliefs about their illness that often do not match medical views but that nevertheless determine, to a large extent, how people respond to their illness. [15] Illness perception is not only based on symptoms, but also on illness related consequences, past experiences of illness and associated anxiety. [16] Research suggests, that patients cluster their ideas about an illness around five coherent themes or components. These components together make up the patient’s perception of their illness. The components provide a framework for patients to make sense of their symptoms, assess health risk, and direct action and coping. Each of these components holds a perception about one aspect of the illness and together they provide the individual’s coherent view of an illness. The components include the timeline of the disease, the ability to control the disease and the extent to which the treatment helps in controlling the disease, the understanding of the cause, and the consequences and the emotional response to them. It also includes the perception of symptoms and the labels attributed to the disease (identity) [15, 17, 18].

Illness perception has a significant implication for adaptation to illness and has been shown to outweigh the impact of medical disease status on depression, physical function and pain and play an important role in the explanation of distress outcome in chronic illness including rheumatic disease [19–21]. It influences how anxious or depressed a patient becomes and these effects may be moderated by other factors such as levels of social support and the presence of additional co-morbidities [22]. In earlier studies, patients’ beliefs about their RA have been related to outcomes such as disability, psychiatric morbidity and pain when clinical measures have not [23]. In chronic disease, cognitive therapies are partly directed at influencing illness beliefs, they are thus amenable to interventions [24].

We assume that, in RA patients, specific beliefs in the timeline of the disease, the ability to control the treatment, the consequences, the comprehensibility of the disease and the emotional response are related to physical and mental HRQoL. Thus, in the present study, we aimed to analyse the association of these illness perceptions with physical and mental HRQoL in patients with RA.

## Material and methods

### Study design and sample

This cross-sectional study was conducted in the rheumatology department at the Kaiser Franz Josef Hospital in Vienna, Austria. RA patients were consecutively screened by their rheumatologist for eligibility during regular outpatient visits. Patients were considered eligible to participate if they had a confirmed diagnosis of RA and a stable disease course with an RA drug regimen for at least 3 months prior to entering the study. Participants had to be ≥18 years old, had to live at home independently and had to be both physically and mentally able to read, understand and complete the questionnaires. Patients were excluded if they had participated in another study and if they had any significant co-morbidity (e.g. advanced cancer or serious mental health problems).

The study was conducted in accordance with the Helsinki Declaration. Ethical review committee approval was obtained from the city government of Vienna (ERB no.: EK-13-190-VK). Participants received detailed information about the study and written informed consent was obtained from each patient before enrolment.

### Measures

Patients were addressed during their regular visits at the outpatient department. A questionnaire package comprising socio-demographic and clinical data, and social support, quality of life and illness perception items was administered to, and completed by, each participant. The socio-demographic variables were sex, age, marital status, educational level and monthly income. Clinical data included disease duration, current RA treatment, the presence of co-morbidities and the number of routine medications (other than RA medication). The subjective perception of current disease activity, pain, fatigue and functional disability was measured on a visual analogue scale, resulting in a score between 0 and 100.

### Health-related quality of life

The Medical Outcome Study 36-item Short-Form Health Survey (SF-36) [25, 26] is an internationally frequently used instrument for operationalizing HRQoL and has been used for various diseases and in many different health-care settings. It measures HRQoL as the extent to which physical health influences an individual’s functional ability and perceived emotional well-being. In RA, the SF-36 has been found to be a reliable and valid instrument, correlating well with disease-specific measures [27]. For our Austrian outpatient population, we used the validated German version of the SF-36 [28].

The SF-36 has eight dimensions comprising limitations in physical activities due to health, social limitations due to physical and emotional problems, limitations in usual role activities due to physical problems, role limitations due to emotional problems, bodily pain, general mental health, vitality and general health perception. Answer formats range from two- to six-point scales and subscales are summed scores that are converted to percentages. Subscale scores can be combined into two summary scores, one for the physical component (assessing role functioning due to physical problems, general physical function, vitality, pain and general health perception) and one for the mental component (assessing social and role functioning, vitality, mental health and general health perception). The scores are calculated by weighting the four subscales positively in the physical domain and negatively in the psychological domain subscales for the Physical Component Summary (PCS) and the reverse for the Mental Component Summary (MCS). Scores range from 0 to 100, with higher scores indicating better functioning [25, 26].

### Illness perception

Based on Leventhal’s Common-Sense Model [15], the Illness Perception Questionnaire (IPQ) was designed to assess underlying dimensions of patients’ perception of their illness. The instrument has good psychometric properties and has been used in a variety of illnesses including RA [29, 30]. For this study, a revised, −RA-specific version of the questionnaire was used (IPQ-R) [29]. The IPQ-R was translated and validated in German [31].

The IPQ-R contains nine scales representing the dimensions underlying the patients’ individual perceptions. The scales and implications of the IPQ-R are listed in Table 1.

**Table 1:** Means (and standard deviations) across illness perception (IPQ-R), social support (SSUK) and health- related quality of life (SF-36) scales and description of scores

	Items in scale/ Ref Range	Mean (SD)	Min–max	Higher score implies
Illness perception				
Identity	14 / 0–14	4.49 (2.54)	1–13	More symptoms experienced and attributed to RA
Timeline (acute/chronic)	5 / 5–25	20.48 (3.53)	6–25	Stronger belief in chronic course of RA
Consequences	5 / 5–25	15.00 (5.25)	5–24	Greater perceived influences of RA
Treatment control	4 / 4–20	13.53 (2.66)	5–20	Greater perceived control by RA treatment
Personal control	4 / 4–20	12.64 (3.32)	4–20	Greater perceived personal control
Illness coherence	5 / 5–25	15.88 (4.21)	5–25	A better understanding of the illness
Timeline (cyclical)	4 / 4–20	13.53 (3.30)	6–20	Stronger belief in repeated recurrence of RA
Emotional representation	5 / 5–25	14.74 (5.16)	5–25	Greater feelings of concern about RA
Social support				
Positive support	4 / 0–16	12.7 (3.2)	4–16	Higher level of positive social support
Negative interaction	4 / 0–16	5.5 (3.7)	5–16	Higher level of detrimental social interactions
Health-related quality of life				
Physical quality of life Mental quality of life	/	38.38 (9.76)	13.84–57.34	Better physical quality of life
	/	46.94 (12.53)	13.89–68.98	Better mental quality of life

*RA* rheumatoid arthritis

The *identity scale* provides a measure of the perceived symptom load with a high score indicating a greater number of symptoms. It is assessed by asking the participants which of 14 symptoms they have experienced during their RA and to identify the symptoms they feel are related to their RA. A high score on the *personal control scale* and *treatment control scale* indicates a greater perceived personal control of the disease and a greater perceived control by medical treatment, respectively. *The timeline acute/chronic* and *timeline cyclical scales* reflect the assumption about chronicity and recurrence of the illness. A higher score on the *consequences scale* implies a belief in a stronger influence of the disease upon everyday life and a higher score on the *coherence scale* indicates a better comprehension (understanding) of the disease. A stronger negative emotional response to the illness is indicated by a higher score on the *emotional representation scale*. The *causes scal*e refers to the causal beliefs about RA. As this scale simply indicates which causal attributions they endorse, it is statistically not useable and was thus not included in the study.

The identity scale is scored by summing the symptoms each patient experienced during, and attributed to, the illness. The remaining scales are rated by the patient on a five-point scale ranging from ‘strongly disagree’ to ‘strongly agree’. After reverse scoring, items are then summed to the single category scores.

### Social support

The eight-item Illness-specific Social Support Scale (SSUK-8) was used to assess the subjective perception of social support in chronically ill patients. It is the brief form of the validated German adaptation of the illness-specific Social Support Scale (SSUK). Items are scored on a five-point Likert scale, converted and summed up into two subscales: positive support and detrimental interactions. Scores range between 1 and 4, with higher scores on each scale indicating higher levels of positive support and higher levels of detrimental interactions, respectively [32–34].

### Statistical methods

Data were analysed using IBM SPSS Version 24.0 for Windows. Descriptive statistics were used to describe demographic and clinical patient characteristics. Categorical variables are presented as N and percentage of total study population. Mean and standard deviation were used for describing continuous variables including illness perception, social support, and physical and mental quality of life. Exploratory linear regression models were used to assess the relationships between outcome variables (illness beliefs, social support) and potential covariates (demographic and clinical factors). Variables that retained independent associations with outcome at univariate level (*p*-value < 0.2 ) were considered for multivariate analysis. Demographic and clinical characteristics were used as categorical variables. The different types of RA medication were included as dichotomous variables (yes/no) and illness perception, HRQoL and social support were included as continuous variables as listed in Tables 2 and 3. All *p*-values are two-sided. Standardized βs were used to compare the strength of independent variables.

**Table 2:** Patient and clinical characteristics

Variable	Total population *N* = 120 (%)
Sex	
Female	99 (82.5)
Male	21 (17.5)
Age	
< 45 years	29 (24.2)
45–65 years	65 (54.2)
> 65 years	26 (21.7)
Marital status	
Single	16 (13.3)
Married	69 (57.5)
Divorced/living separated	24 (20.0)
Widowed	11 (9.2)
Educational level	
Compulsory/lower secondary	31 (25.8)
Secondary school/apprenticeship	68 (56.7)
Higher education	9 (7.5)
Other	12 (10.0)
Monthly net income	
< 1.000€ (1120$)	23 (19.2)
1.001–2000 € (1121–2240 $)	68 (56.7)
2001–3000 € (224—3360 $)	20 (16.7)
> 3000€ (> 3361$)	9 (7.5)
Disease duration	
> 10 years	35 (42.5)
5–10 years	40 (33.3)
1–4 years	34 (30.8)
< 1 year	8 (6.7)
RA treatment	
NSAID	51 (42.5)
sDMRAD	106 (88.3)
bDMRAD	38 (31.7)
Steroid	29 (24.2)
Other	11 (9.2)
Additional medication	
No other medication	34 (28.3)
1–2 other medications	45 (37.5)
3–4 other medications	19 (37.5)
> 4 other medications	22 (18.3)
Presence of co-morbidity	62 (51.7)
	Mean (SD)
Patient self–assessed (VAS)	37.8 (30.3)
Disease activity	34.7 (29.4)
Pain	40.8 (29.0)
Functional disability	38.3 (31.0)
Fatigue	

*NSAID* non–steroidal anti–inflammatory drug, *sDMRAD* synthetic disease–modifying antirheumatic drug, *bDMARD* biologic disease–modying antirheumatic drug, *VAS* Visual analogue scale 0–100

**Table 3:** Univariate analysis of associations between patient and clinical characteristics, illness perception, social support and HRQoL

	Physical quality of life	*P*-value	Mental quality of life	*P*- value
Patient characteristics				
Sex	−0.06	0.500	0.01	0.940
Age	−0.13	0.152	0.12	0.177
Marital status	−0.15	0.100	0.09	0.323
Educational level	0.14	0.122	0.07	0.429
Monthly net income	0.11	0.256	0.14	0.121
Clinical characteristics				
Disease duration	−0.06	0.550	0.16	0.078
Treatment				
NDAID	0.46	< 0.001	0.22	0.017
sDMARD	0.05	0.624	0.06	0.517
bDMARD	−0.08	0.405	0.01	0.942
Steroid	0.29	0.001	0.31	< 0.001
Additional medication	−0.34	< 0.001	− 0.28	0.002
Co-morbidity	−0.31	< 0.001	− 0.34	< 0.001
Disease activity	−0.56	< 0.001	−0.54	< 0.001
Pain	−0.56	< 0.001	−0.56	< 0.001
Functional disability	−0.56	< 0.001	− 0.61	< 0.001
Fatigue	−0.48	< 0.001	− 0.64	< 0.001
Illness perception				
Identity	−0.39	< 0.001	− 0.39	< 0.001
Timeline (acute/chronic)	−0.11	0.228	0.03	0.719
Consequences	−0.61	< 0.001	−0.56	< 0.001
Treatment control	0.22	0.014	0.20	0.029
Personal control	0.29	0.001	0.31	0.001
Illness coherence	0.23	0.012	0.42	< 0.001
Timeline (cyclical)	−0.58	< 0.001	− 0.38	< 0.001
Emotional representation	−0.39	< 0.001	−0.60	< 0.001
Social support				
Positive support	0.06	0.553	0.16	0.085
Detrimental interaction	−0.31	0.001	−0.41	< 0.001

Values shown are beta regression coefficients based on univariate regression analyses with dependent variable physical/mental HRQoL

## Results

### Sample characteristics

A total of 120 patients were included and completed the questionnaires. The demographic and clinical sample characteristics are presented in Table 2. The majority of the included patients were female. The mean age was 54.2 (SD = 12.9) and most of the patients had at least secondary school education. Most patients were either currently employed or permanently retired, had a monthly net income of between 1001 and 2000 € (1121–2240$) and were either married or living in a stable relationship. Disease duration was quite evenly distributed among patients, with only eight participants having a disease duration of less than 1 year (inclusion criteria: a stable disease and therapy). Most patients were on a therapy with synthetic disease-modifying antirheumatic drugs (sDMARDs) and one-third were receiving biological disease-modifying antirheumatic drugs (bDMARDs). More than half of the patients suffered from at least one co-morbidity and the majority of patients took at least one additional routine medication (other than RA medication).

### Physical health-related quality of life

Univariate analysis (Table 3) revealed that the variables age, marital status, educational level, monthly net income, number of additional medications, presence of co-morbidity, pain, disease activity, functional disability, fatigue, detrimental social interaction and all dimensions of illness perception except timeline (acute/chronic) were significantly associated with physical HRQoL at the *p* <  0.2 level and were therefore included in the multivariate analysis (Table 4). We did not include NSAID and steroid treatment due to multicollinearity with pain and disease activity. As shown in the crude regression model, which is not adjusted for possible confounders (Table 4, Model 1), *consequences* and *timeline cyclical* were associated with physical HRQL. This association remained stable when adjusted for demographic and clinical confounders and social support. These results indicate that RA patients with stronger belief in the influence of RA on their daily life and stronger belief in the chronicity of the disease have lower physical HRQoL. In the multivariate analysis, none of the demographic characteristics, clinical parameters and social support were significantly associated with physical HRQoL.

**Table 4:** Regression models between illness perception items, patient characteristics, social support and dependent variable physical health-related quality of life (SF-36)

	Model 1 (Block 1)		Model 2 (Block 1–2)		Model 3 (Block 1–3)		Model 4 (Block 1–4)	
	R^2^	β	R^2^	β	R^2^	β	R^2^	β
Block 1								
Illness perception	0.511		0.536		0.565		.0.565	
Identity		−0.07		−0.08		−0.05		−0.05
Consequences		− 0.37^**^		−0.39^**^		− 0.34^**^		−0.34^**^
Treatment control		0.04		0.06		0.04		0.04
Personal control		0.14		0.08		0.03		0.03
Illness coherence		0.01		−0.04		−0.04		−0.05
Timeline (cyclical)		−0.39^**^		−0.36^**^		−0.30^**^		−0.30^**^
Emotional representation		−0.05		−0.02		− 0.01		- 0.00
Block 2								
Demographic characteristics								
Age				−0.16		− 0.16		− 0.16
Marital status				−0.06		−0.08		− 0.08
Educational level				−0.04		− 0.07		−0.07
Block 3								
Clinical characteristics								
Additional medication						0.03		0.03
Co-morbidity						−0.08		−0.08
Disease activity						−0.03		−0.03
Pain						0.01		0.01
Functional disability						−0.18		−0.18
Fatigue						−0.02		−0.02
Block 4								
Social support								
Detrimental interaction								0.08
R^2^ change			0.53			0.56		0.63

*p ≤ 0.05;** *p* ≤ 0.01; R^2^ is adjusted R^2^; β is standardized coefficient β

All variables with a *p*-value < 0.2 in univariate analysis were included in the regression model

### Mental health-related quality of life

Univariate analysis (Table 3) revealed that the variables age and monthly net income, number of additional medications, presence of co-morbidity, pain, disease activity, functional disability, fatigue, positive as well as detrimental social interactions and all dimensions of illness perception except timeline (acute/chronic) were significantly associated with mental HRQoL at the *p* <  0.2 significance level and were therefore included in the multivariate analysis (Table 5). In the regression model, which is not adjusted for possible confounders (Table 5, Model 1), consequences, illness coherence and emotional representation were associated with mental HRQL. When adjusted for clinical characteristics (Model 3), only illness coherence and emotional representation were associated with mental HRQoL, was along with fatigue. After additional adjustment for social support (Model 4), only emotional representation and fatigue remained significantly associated.

**Table 5:** Regression models between illness perception items, patient characteristics, social support and dependent variable mental health-related quality of life (SF-36)

	Model 1 (Block 1)		Model 2 (Block 1–2)		Model 3 (Block 1–3)		Model 4 (Block 1–4)	
	R^2^	β	R^2^	β	R^2^	β	R^2^	β
Block 1								
Illness perception	0.452		0.447		0.554		0.582	
Identity		−0.12		−0.12		−0.02		−0.01
Consequences		−0.22^*^		− 0.21^*^		− 0.07		−0.03
Treatment control		−0.02		−0.03		− 0.03		− 0.01
Personal control		0.07		0.09		0.03		0.01
Illness coherence		0.19^*^		0.19^*^		0.18^*^		0.12
Timeline (cyclical)		−0.06		−0.07		0.04		0.03
Emotional representation		−0.34^**^		−0.31^**^		−0.26^*^		−0.27^*^
Block 2								
Demographic characteristics								
Age				0.08		0.10		0.08
Monthly net income				0.02		0.03		0.02
Block 3								
Clinical characteristics								
Additional medication						−0.02		−0.02
Co-morbidity						−0.09		−0.07
Disease activity						0.08		0.13
Pain						−0.12		−0.15
Functional disability						−0.10		−0.11
Fatigue						−0.33^**^		−0.36^**^
Block 4								
Social support								
Positive support								0.19
Detrimental interaction								−0.10
R^2^ change				0.43		0.55		0.58

*p ≤0.05;** *p* ≤ 0.01; R^2^ is adjusted R^2^; β is standardized coefficient β

All variables with a *p*-value < 0.2 in univariate analysis were included in the regression model

## Discussion

RA has a substantial effect on HRQoL [35], and routine assessment of the disease’s impact on a patient’s life, including assessment of fatigue and HRQoL, has been recommended in recent guidelines [36]. In the present study, we assessed physical and mental HRQoL in an outpatient population of RA patients with stable disease course and examined illness perception and symptoms attributable to RA and their contribution to physical and mental HRQoL (SF-36).

The main finding of this study was, that in multivariate analysis, only two aspects of illness perception (consequences and cyclical timeline) explained 63% variance in physical HRQoL and only one aspect of illness perception (emotional representation) and fatigue explained 58% variance in mental HRQoL.

Our study supports the CSM, which states that people’s beliefs about their illness are associated with their HRQoL. Although there are studies showing that the relation between illness perception and illness outcomes, including overall well-being, are mediated by coping with illness [37], there is evidence, that in chronic disease, illness perception has a stronger influence on HRQoL than coping strategies [38, 39].

In our study perceived little impact on daily life (consequences) and a weaker belief in the recurrence of RA (timeline cyclical) was associated with a higher score in physical HRQoL. In a qualitative study on fears and beliefs in rheumatoid arthritis the unpredictability of the disease course was a common source of anxiety, however, these fears were lower in remission but when exacerbations occurred, or a treatment stopped working initial fears were reactivated. The variability in the course was in itself a major source of uncertainty and worries. Also, no longer being able to manage activities of daily living was a common fear on the impact of the disease. This covered all aspects of daily living from getting up to driving or climbing stairs [40].

Our finding that patients with a weaker emotional response to their disease is associated with a better mental HRQoL is in line with previous studies on RA [41] and other chronic diseases [42].

Fatigue had a significant impact on mental HRQoL in our fully adjusted model. A literature review [2] reported that unacceptable levels of fatigue persisted in a substantial proportion of patients with RA, despite the introduction of intensive treatments and the fact that a majority of patients with RA believed that reducing fatigue should be a key treatment aim, although fatigue-related end points were rarely reported in clinical trials.

In a study comparing the relative predictive value of disease status and illness perception, clinical variables explained some but illness perception explained the biggest part of the variance in disease-specific functioning [43]. Also, in our population in univariate regression, demographics and the clinical parameters co-morbidity, number of co-medications, disease activity, functional disability, pain and fatigue were significantly associated with physical and mental HRQoL, but when adapting these variables in the multivariate regression with illness beliefs, only fatigue remained a significant contributor to mental HRQoL.

The mean physical SF-36 score reported in this study was 38.38 and the mean mental SF-36 summary score was 46.94.

These findings are in line with a previous study and a review [6, 35], that reported similar score levels for the RA population compared to significantly higher levels in the general population.

Notably, a higher age was associated with a reduced physical HRQoL but higher mental HRQoL, supporting two previous reviews that reported positive associations between age and mental HRQoL as well as negative associations between the prevalence of depression and age in RA [35, 44].

Here the question arises, if a chronic disease like RA has a stronger impact on younger patients, as it is more difficult for them to accept the limitations caused by the disease (e.g. loss of workability).

Illness coherence was associated with mental HRQoL in univariate and multivariate regression before adjustment to social support, suggesting that people with better knowledge of their disease have a better mental HRQoL. Low health literacy was shown to be associated with functional impairments and disease severity [45] but has until now been reported in association with HRQoL in RA. The better patients knew their disease, the better they experienced mental HRQoL, presumably due to the security of knowing the disease and what to expect in the course of disease and treatment. To the best of our knowledge, the positive impact of better disease understanding on QoL has not been studied in RA patients before and might be interesting for future studies.

When interpreting this study’s results, several points should be taken into consideration.

First, a theoretical concern is the conceptual overlap between specific illness perceptions, disease parameters and HRQoL: E.g. ‘identity’ overlaps with subjectively perceived disease activity/pain (VAS scales). Here the use a standard tool to assess disease activity would cause less overlap. ‘Consequences’ overlaps with physical aspects and emotional representation with emotional aspects of quality of life scales. These overlaps have been already discussed e.g. in a study on patients with inflammatory bowel disease [46]. Also, according to cognitive behavioural theory, we hypothesized that a trigger (RA) leads to a cognitive process (illness perception) which influences emotional and behavioural factors (HRQoL). It is however possible, that in turn poor HRQoL due to other factors/circumstances leads to more negative illness perceptions.

Second, this study is limited due to its cross-sectional and monocentric character, which does not allow final conclusions to be drawn about the direction and causality of the impact of illness perception on HRQoL. The present study assesses how current illness perception is associated with the current HRQoL in RA patients. The fact that (a singular assessment of) illness perceptions only represent how the patient currently thinks, feels and understands about his/her medical condition and do not portray illness perception as an independent psychological factor, trait or process has to be taken into account [47]. Illness perception is dynamic, can change over time and is amenable to intervention [48], thus a causal relationship can only be verified through a longitudinal study potentially including psychological interventions. Illness perception-focused intervention and its effect on quality of life has been studied, for example, for patients with myocardial infarction and their spouses [49, 50] but not for RA, and is thus an interesting objective for an intervention study on RA. Third, the study population primarily consisted of female participants, reflecting the characteristic population distribution of RA patients, but also features the advantage of a balanced distribution of age and disease duration indicating a representative group of RA patients. Also, the study population was limited to participants with a stable disease course for at least 3 months, which excluded e.g. patients with acute disease flares. Patients with currently unstable disease might report other dimensions of illness perception and associations to HR QoL might differ broadly. Thus, these findings are possibly not generalizable to the entire RA population. Fourth, disease activity was assessed using a self-reported parameter and not by means of a recognized objective assessment tool such as the clinical disease activity index and may therefore not be comparable to other study results.

Despite these limitations, this study provides additional information to determinants of HRQoL in RA patients. To our knowledge multiple studies exist on the relation on illness perception on e.g. depression, subjective disease assessment or disability but only few to measure the relation of illness perception of HRoL in RA.

In RA, beliefs about the disease appear to evolve over the course of the disease [40] and illness perceptions are well defined as target to treatment in cognitive behavioural therapy[51]. A substantial finding of this study was, that in patients with stable RA illness perceptions were associated with HRQoL rather than clinical parameters. Given the low HRQoL in RA patients, beside pharmaco-, and physiotherapy, a psychological support could be added for a complete treatment approach. To our knowledge no interventional study was published on illness perception focused therapy on RA patients yet [52]. The results of this study suggest, that targeting illness perception could be tested in psychological interventions to improve HROoL, which has been shown e.g. for musculoskeletal pain [53].

## Conclusion

This study showed that current illness perception accounts for a large part of the variance in physical and mental health-related quality of life after adjusting for demographics, clinical variables and social support. Although a longitudinal study with illness perception intervention is needed to assess the stability of beliefs and associated changes in HRQoL, the present study showed that the characterization of a patient’s illness perception is associated with his/her HRQoL. The question if HRQoL can be positively influenced by alleviating the perceived threat of their illness and strengthening positive illness beliefs would be an interesting matter for future research.

## Abbreviations

CSM: Common-sense model
HRQoL: Health-related quality of life
IPQ: Illness perception questionnaire
IPQ-R: Revised, RA-specific version of IPQ-R
MCS: Mental component summary
PCS: Physical component summary
RA: Rheumatoid arthritis
SF-36: Medical outcome study 36-item short-form health survey
SSUK-8: Eight-item illness-specific social support scale
